# The Psychological Impact of COVID-19 on Front Line Nurses: A Synthesis of Qualitative Evidence

**DOI:** 10.3390/ijerph182412975

**Published:** 2021-12-09

**Authors:** Sara Huerta-González, Dolores Selva-Medrano, Fidel López-Espuela, Pedro Ángel Caro-Alonso, Andre Novo, Beatriz Rodríguez-Martín

**Affiliations:** 1 Faculty of Nursing, Universidad Veracruzana, Tuxpan, Poza Rica 91000, Mexico; sahuerta@uv.mx; 2 Health Service of Castilla-La Mancha, University Hospital Complex of Albacete, C. Hermanos Falco, 37, 02006 Albacete, Spain; dselva@sescam.jccm.es; 3 Metabolic Bone Diseases Research Group, Department of Nursing, Faculty of Nursing and Occupational Therapy, University of Extremadura, 10004 Caceres, Spain; 4 Faculty of Health Sciences, University of Castilla-La Mancha, Avd/Real Fábrica de Sedas s/n, 45660 Talavera de la Reina, Spain; PedroA.Caro@uclm.es (P.Á.C.-A.); Beatriz.RMartin@uclm.es (B.R.-M.); 5 Instituto Politécnico de Bragança, 5300-253 Bragança, Portugal; andre@ipb.pt

**Keywords:** COVID-19, nurse, psychological distress, qualitative research, systematic review

## Abstract

Caring for people with COVID-19 on the front line has psychological impacts for healthcare professionals. Despite the important psychological impacts of the pandemic on nurses, the qualitative evidence on this topic has not been synthesized. Our objective: To analyze and synthesize qualitative studies that investigate the perceptions of nurses about the psychological impacts of treating hospitalized people with COVID-19 on the front line. A systematic review of qualitative studies published in English or Spanish up to March 2021 was carried out in the following databases: The Cochrane Library, Medline (Pubmed), PsycINFO, Web of Science (WOS), Scopus, and CINHAL. The PRISMA statement and the Cochrane recommendations for qualitative evidence synthesis were followed. Results: The main psychological impacts of caring for people with COVID-19 perceived by nurses working on the front line were fear, anxiety, stress, social isolation, depressive symptoms, uncertainty, and frustration. The fear of infecting family members or being infected was the main repercussion perceived by the nurses. Other negative impacts that this review added and that nurses suffer as the COVID-19 pandemic progress were anger, obsessive thoughts, compulsivity, introversion, apprehension, impotence, alteration of space-time perception, somatization, and feeling of betrayal. Resilience was a coping tool used by nurses. Conclusions: Front line care for people with COVID-19 causes fear, anxiety, stress, social isolation, depressive symptoms, uncertainty, frustration, anger, obsessive thoughts, compulsivity, introversion, apprehension, impotence, alteration of space-time perception, somatization, and feeling of betrayal in nurses. It is necessary to provide front line nurses with the necessary support to reduce the psychological impact derived from caring for people with COVID-19, improve training programs for future pandemics, and analyze the long-term impacts.

## 1. Introduction

The first reported case of COVID-19 emerged in Wuhan, China in late 2019 [1]. The virus that causes this disease is SARS-Co-V2, from the family of coronaviruses that are the origin of the most virulent diseases in humans, along with MERS (Middle East Respiratory Syndrome) and SARS (Severe Acute Respiratory Syndrome) [2]. Since then, COVID-19 has spread throughout the world, reaching pandemic dimensions, and having serious health, economic, and social impacts. As of 17 September 2021, the number of infected people in the world was 219 million, and 4.55 million deaths have been reported from this disease [3].

The most common symptoms of COVID-19 are fever, dry cough, tiredness, loss of appetite, confusion, chest tightness or pain, and dyspnea. Complications of this disease that can lead to death include respiratory failure, acute respiratory distress syndrome, sepsis and septic shock, thromboembolism, and multiple organ failure [3,4]. In addition, some of the people affected with COVID-19 continue to experience long-term respiratory and neurological symptoms or fatigue [3].

Since the start of the pandemic, nurses have been on the front line caring for people with COVID-19. Various quantitative and qualitative studies show the impact of working on the front line with people with COVID-19, highlighting the following psychological impacts: anxiety, stress, depression [5,6], post-traumatic stress syndrome, psychological distress, and mental exhaustion [7]. In addition, previous studies show that the risk of being infected, the fear of infecting [8], social stigma [9], the discomfort of working with personal protective equipment (PPE) [10], and social isolation or uncertainty [11] are associated with increased stress and anxiety in professionals working on the front line [5,12]. Impacts are aggravated when there is no adequate emotional support in the work environment, there are insufficient security measures, or there is no adequate institutional support [13].

On the other hand, certain studies have looked at factors that increase the risk of developing mental health problems among front line healthcare professionals. Thus, a study highlights the workload, having respiratory or digestive symptoms, having carried out specific diagnostic tests for COVID-19, such as CRP, overseeing a family member, having a negative coping style, or work exhaustion [14]. Other influencing factors also reported are a history of psychiatric illness, working on the front line, not having contracted the disease [15], having an underlying organic pathology, being a woman, expressing concern for the family, fear of infection, lack of PPE [16], or being a nurse [7,17]. Concerning this last factor, we know that nurses working on the front line are significantly more likely to experience anxiety symptoms than other healthcare professionals [18], higher levels of stress and subjective load [19], and higher scores on insomnia and distress scales [20].

Several studies analyze the psychological manifestations and perceptions of health professionals in the first line, but most are quantitative, without inquiring into perceptions, or do not distinguish between professional categories [12,21,22].

There are two previous qualitative systematic reviews published in 2020 that analyze the experiences of nurses working in hospital settings caring for people with COVID-19 [23,24], but none of them aim to analyze the psychological impacts on nurses. One of them includes studies since the beginning of the pandemic and of previous respiratory epidemics such as SARS, analyzing, among other categories, the psychological and emotional impact on front line nurses, but it does not exclusively analyze the COVID-19 pandemic, so it cannot be extrapolated to the current situation [23]. The second review’s aim was to analyze the barriers perceived by nurses to care for people with COVID-19, and it only includes articles published between January and August 2020, analyzing only the first part of the pandemic, highlighting among its findings the emotional and psychological stress experienced by front line nurses, in addition to analyzing other aspects such as limited information on COVID-19 and the lack of support from managers perceived by nurses [24].

A large part of the psychological impacts appears in the long term, so it is necessary to carry out a synthesis of the updated qualitative evidence that analyzes the perceptions of nurses about the psychological impacts of caring for people with COVID-19 in the front line, including studies that have analyzed long-term impacts and various waves of the pandemic. Key information is needed that will help provide a global vision of the problem and its evolution as the pandemic progresses and to develop support strategies for front line nurses facing these types of health emergencies.

This review is focused on the perceptions of front line nursing professionals, without including other professionals. Compared to previous reviews, studies that investigate the first, second, and subsequent waves are included, with which the impact in the medium and long term can be analyzed. In addition, this review focuses exclusively on analyzing the psychological impacts, without considering other aspects that affect the nursing care of people with COVID-19.

The objective of this qualitative systematic review is to analyze and synthesize qualitative studies that investigate the perceptions of nurses about the psychological impacts of treating hospitalized people with COVID-19 in the front line.

## 2. Materials and Methods

### 2.1. Protocol and Registration

A qualitative systematic review was carried out according to the Cochrane recommendations for the synthesis of qualitative evidence [25] and the PRISMA 2020 recommendations for systematic reviews [26]. The protocol of this review was registered in PROSPERO (279339).

### 2.2. Eligibility Criteria, Sources of Information, and Search Strategy

A systematic review of qualitative studies published in English or Spanish up to March 2021 was carried out in the following databases: The Cochrane Library, Medline (Pubmed), PsycINFO, Web of Science (WOS), Scopus, and CINHAL. A secondary search was also carried out through the references found in the included articles.

Table 1 shows the search strategy used in the different databases, which was adjusted according to the requirements of each database (Table 1).

The search and selection process of the articles was carried out independently by two researchers according to the established inclusion and exclusion criteria, subsequently agreeing on the results. In case of disagreement, a third reviewer mediated. During this process, the following criteria were used. Inclusion criteria: (1) Qualitative studies that analyzed the perceptions of nurses about the psychological impacts of caring for people with COVID-19 in the front line. (2) Studies carried out in the hospital setting. (3) Studies published in English or Spanish published until March 2021. Exclusion criteria: (1) Systematic reviews. (2) Mixed designs if the qualitative data were not analyzed in a disaggregated way. (3) Studies conducted before the COVID-19 pandemic or looking at other pandemics. (4) Studies that analyzed the perceptions of front line professionals without carrying out a separate analysis by professional category. (5) Studies whose sample included nursing students.

### 2.3. Selection of Studies

**Figure 1:**
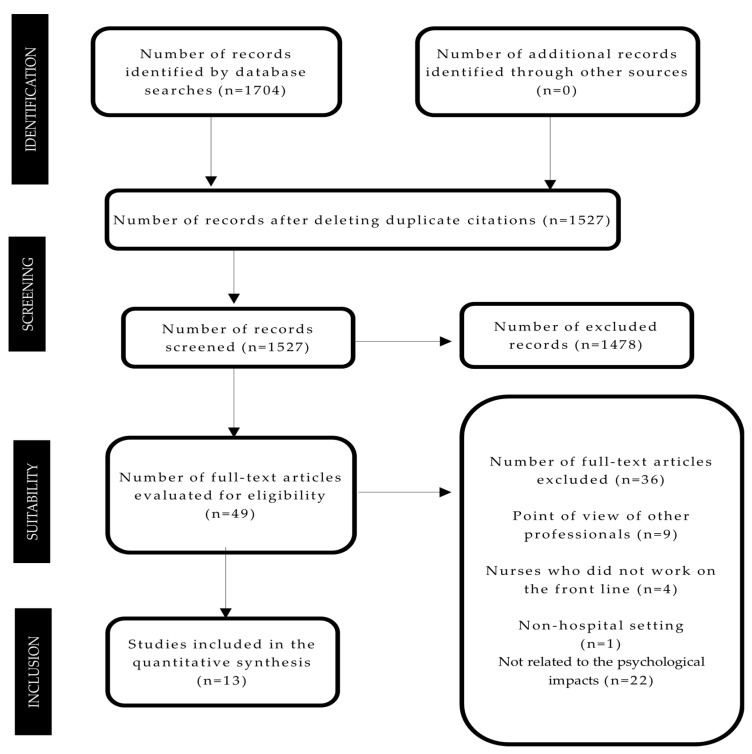
Flow diagram of the study search and selection process.

Screening of eligible studies was conducted in duplicate and independently by two reviewers following the established inclusion and exclusion criteria, in case of disagreement, a third reviewer was used. First, a search was carried out by the title and abstract After that, full-text articles were reviewed. The study selection was made following the flow chart of the study search and selection process that appears in Figure 1.

### 2.4. Data Extraction Process, Data List, and Summary Measures

The data were extracted independently by two researchers who subsequently agreed on the results, using an Excel template that included the data on the main characteristics of the analyzed studies, which are shown in Table 2.

### 2.5. Quality Assessment of Included Studies

The quality of the analyzed studies was assessed using the CASP tool for qualitative studies [40]. This tool was not used for inclusion or exclusion criteria, but rather to provide information on its methodological quality (Table 3).

## 3. Results

### 3.1. Study Selection Process

After searching the databases, 1704 articles were obtained from which duplicates were eliminated. After reading the title and abstract, 49 articles were reviewed in full text, of which 36 that did not meet the established criteria and were excluded. Thirteen articles were finally included in the qualitative synthesis upon meeting the selection criteria (Figure 1).

### 3.2. Characteristics of the Included Studies

Table 2 lists the main characteristics of the included studies. The results table collects the identified themes of each study related to the psychological impacts of caring people with COVID-19 expressed by the nurses. In the description of the results, the term “nurse” is used to refer to professionals of both genders as it is an internationally accepted term.

Concerning the paradigmatic approaches used in the analyzed studies, seven used content analysis [28,29,34,36,37,38,39], one the narrative analysis [32], three the phenomenological-descriptive approach [27,31,35], one the phenomenological-hermeneutic [30], and another the phenomenological approach without specifying the type [33]. In relation to data collection techniques, ten studies used semi-structured interviews [27,30,31,32,33,34,36,37,38,39] and three used in-depth interviews [28,29,35].

All studies used intentional sampling. The total sample of the studies was 239 nurses, mostly women. The studies were conducted in Iran [27,28,29], Turkey [30,31], Canada [32], Italy [33], China [34,36,37,38], the United States of America [35], and Spain [39].

Regarding the length of time since exposure to front line work with COVID-19 patients, two of the articles did not specify when the data were collected or the exposure time [27,32]. In the rest, the exposure time ranged between one week [37] and three months [34]. In most articles, the data was collected between January and May 2020 [28,29,31,33,34,35,36,37,38,39], while in one article, the data was collected in June and August 2020 [30].

### 3.3. Synthesis of Results

The analyzed studies showed that front line care for people hospitalized with COVID-19 produces multiple and varied psychological impacts on nurses, without differences according to the exposure time in the analyzed articles. The psychological impacts most verbalized by the nurses were fear [27,28,29,30,31,32,33,34,35,36,37,38,39], anxiety [27,28,29,30,31,34,38,39], stress [27,29,31,36,37,38], social isolation [31,32,35,36,38,39], depressive symptoms [31,38], uncertainty [32,33,36], anger [32,35], and frustration [32,34,39]. To a lesser extent, they also manifested the feeling of pressure [34], obsessive thoughts [28,31], introversion [31], apprehension [32], impotence [32], alteration of space-time perception [33], helplessness [34], somatization [38], feeling of betrayal [28], feeling overwhelmed [35], mental exhaustion [35], psychological pain [35], compulsivity [38], and sadness [32].

The main fears expressed by the nurses were infecting relatives or being infected [28,29,30,37,38], followed by the fear of death of the patients, both due to the high number of deaths and the conditions in which they occurred [29], fear of infection due to ignorance of the behavior of the virus [36], and the fear of death itself [27] or death of family members [27].

Anxiety also manifested itself in four subcategories: family separation anxiety (29), anxiety about the death of patients [28], anxiety about the nature of the illness [28], and anxiety before the burial of the corpses [28].

Resilience is understood as the ability to adapt flexibly to changes caused by stressful events and recover from negative emotional experiences and was considered by the nurses as the main coping technique to counteract the losses and traumas they experienced [32].

### 3.4. Analysis of the Quality of the Studies

The results of the quality analysis of the included studies were carried out with the CASP tool for qualitative studies and are shown in Table 3. All the articles fulfilled the items on the CASP tool, except for three articles that did not provide information about the relationship between researchers and participants [32,33,34] and another article that did not provide information about the rigor [28].

## 4. Discussion

This is the only qualitative systematic review to explore exclusively front line nurses’ perceptions about the psychological impacts of treating hospitalized people with COVID-19. None of the previous reviews exclusively analyze the psychological impacts on nurses working in hospital settings caring for people with COVID-19 [23,24]. A previous review includes articles from other respiratory epidemics [23] and the aim of another was to analyze the barriers perceived by nurses to care for people with COVID-19 [24]. The main psychological impacts of caring for people with COVID-19 perceived by nurses working on the front line were fear, anxiety, stress, social isolation, depressive symptoms, uncertainty, and frustration. The fear of infecting family members or being infected was the main repercussion perceived by the nurses.

It is known that the COVID-19 pandemic increases the level of anxiety and fear, stress, depression, frustration, and uncertainty among the general population [41,42]. In the case of nurses, the impacts are greater for caring on the front line, and new repercussions appear, which are explained below.

Fear, vulnerability, and psychological distress have been reported in a previous review as the main psychological consequences of front line care in other respiratory epidemics [23]. Our results follow the line of this review, showing that front line care during a pandemic leaves nurses feeling vulnerable, under pressure, stressed, and powerless. Moreover, our results show that nurses experienced loneliness and a high level of anxiety, more than that in the general population, due to concern for their health when caring for patients infected during these pandemics. The fear of infecting their family and friends is also common in nurses and in the general population [23,41,42] and is associated with an increased feeling of loneliness [23]. 

Furthermore, the results of this review are consistent with those of a previous review looking at studies conducted during the first two waves of the COVID-19 pandemic [24], showing that the main concern expressed by nurses working on the front line is transmitting the virus to their family members. In addition, our results also coincide with this review, contributing other psychological impacts of caring in the front line, such as feelings of anxiety, fear, overwhelm, mental exhaustion, depression, helplessness, and the social isolation experienced by nurses. Other psychological impacts perceived by nurses that do not appear in previous reviews [23,24] include anger, obsessive thoughts, compulsivity, introversion, apprehension, impotence, alteration of space-time perception, somatization, and feeling of betrayal.

The psychological impacts suffered by professionals working on the front line have been analyzed in multiple quantitative studies [7,11]. Our results are in line with those of a quantitative systematic review [43] which analyzes the psychological impacts of other pandemics and highlights that fear is considered by professionals as the main psychological manifestation along with anxiety and depression. In contrast, other quantitative reviews that analyze the first waves of the pandemic find that stress, anxiety, and depression are the main impacts of front line care [7,44]. These differences may be because this quantitative review analyzes the early psychological impacts of front line professionals, which can be sustained over time, while new symptoms appear as the pandemic evolves. As previously noted [45], the initial stress that professionals are subjected to in the face of the COVID-19 pandemic can evolve and manifest itself in different ways while healthcare personnel is exposed to this health emergency. Our findings follow this line, although more studies would be necessary to confirm whether these changes are maintained during the different stages of the pandemic.

The nurses in the studies included in this review considered that resilience helped counteract the losses and trauma they experience. According to a recent review, resilience can be improved by providing adequate information, psychosocial support, assigning tasks and responsibilities according to their intensity, and creating adequate working conditions [46].

Other studies suggest that improving the psychological assessment of professionals on an ongoing basis [47], improving work shifts and reducing workload [48], encouraging self-care [49], maintaining effective communication with workers, involving mental health professionals in supporting nurses, and ensuring the provision of personal protective equipment [50] are effective measures to reduce the psychological impact on front line health workers during the pandemic.

### 4.1. Implications for Clinical Practice

The results of this review show that it is necessary to monitor the psychological impacts in front line nurses [32], implement intervention strategies and psychological counseling [28,29,30,31,37], offer training programs that enable nurses to be better prepared [30], cope with the death of patients [28], and help nurses adapt to the sudden and extreme demands for future pandemics [27]. Providing nurses with enough personal protective material and adequate infrastructure and work environment is essential to reducing their fear [28,29,32]. Moreover, work should be organized based on individual roles and responsibilities [30], and the workforce should be reinforced to reduce workloads [32]. 

### 4.2. Limitations and Strengths

The limitations inherent to systematic reviews such as publication or selection bias should be considered. Furthermore, only including articles published in English or Spanish in the selected databases potentially left out relevant studies.

As a review of qualitative studies, this review provides richness in terms of the categories related to the main psychological manifestations. Future studies should include articles that analyze the long-term psychological impacts experienced by nurses in different waves to see if there are variations over time and include articles that analyze the point of view of nurses who work in different professional fields to find out if there are differences.

Future studies also need to investigate the most effective strategies to reduce the psychological impact on front line nurses to establish the appropriate measures from the onset of future pandemics.

## 5. Conclusions

The results of the study show that the main psychological impacts of caring for people with COVID-19 manifested by the nurses were fear, anxiety, stress, social isolation, depressive symptoms, uncertainty, and frustration. The fear of infecting family members or being infected was the main impact perceived by the nurses. As the pandemic progresses, fear prevails over stress, which was the main psychological impact in the initial phases of the pandemic. Moreover, other negative impacts that nurses suffer as the COVID-19 pandemic progress were anger, obsessive thoughts, compulsivity, introversion, apprehension, impotence, alteration of space-time perception, somatization, and feeling of betrayal. Resilience was considered as the main tool to face losses and trauma experienced by nurses. It is necessary to provide front line nurses with the necessary support to reduce the psychological impact derived from caring for people with COVID-19.

## Figures and Tables

**Table 1:** Search strategy in the selected databases.

Database	Search Chain
PUBMED	(COVID-19 or coronavirus or SARS-CoV-2) and (nurs * or front line health or healthcare providers or healthcare workers) and (qualitative research)
COCHRANE	(COVID-19 or coronavirus or SARS-CoV-2) and (nurs * or front line health or healthcare providers or healthcare workers)
Web of Science	(COVID-19 OR coronavirus OR SARS-CoV-2) AND (nurs * OR front line health OR healthcare providers OR healthcare workers) AND (qualitative research)
CINALH	(COVID-19 or coronavirus or SARS-CoV-2) and (nurs * or front line health or healthcare providers or healthcare workers) and qualitative research
SCOPUS	(COVID-19 OR SARS-CoV-2 OR coronavirus) AND (nurs * OR front line health OR healthcare providers OR healthcare workers) AND (qualitative research)
PSICINFO	(COVID-19 or coronavirus or SARS-CoV-2) and (nurs * or front line health or healthcare providers or healthcare workers)

* It is the truncation in the searches.

**Table 2:** Summary of the main characteristics of the analyzed studies.

Authors (Year), Country	Location	Sample	Methodology	Results	Conclusions
Karimi Z, Fereidouni Z, Behnammoghadam M, Alimohammadi N, Mousavizadeh A, Salehi T, et al. (2020) [27], Iran	Hospital (without specification).	Intentional sample. Twelve nurses (8 women and 4 men) who work in the COVID-19 unit. Average work experience: 6.75 years.	Descriptive phenomenology. Semi-structured interviews. It is not specified when data were collected.	Caring for people with COVID-19 caused psychological impacts on the nurses, highlighting stress, anxiety, and fear of the death of the patients, their own, or that of their relatives.	Nurses consider that caring for people with COVID-19 affects them psychologically, highlighting anxiety, stress, and fear as the main psychological impacts. Policymakers and managers must develop plans that help nurses adapt to the sudden and extreme demands of caring for people with COVID-19. Future research should analyze nursing care in the context of COVID-19 to strengthen care.
Galehdar N, Kamran A, Toulabi T, Heydari H. (2020) [28], Iran	Public hospital.	Intentional sample. Twenty nurses (five men and 15 women) who work in the Emergency Department or Intensive Care Units (ICU) with people with COVID-19. Average experience: 7.25 years.	Content analysis. In-depth telephone interviews conducted between March and May 2020.	Caring for people with COVID-19 caused the nurses anxiety (in the face of death, the nature of the disease, or the burial of the corpse), fear (of infecting family members or being contaminated), and the appearance of obsessive thoughts.	Nurses caring for people with COVID-19 perceive that they have the following psychological impacts: anxiety, fear, and obsessive thoughts. Implementing educational programs on death for nurses who care for people with COVD-19 could reduce anxiety and improve the quality of care. Providing nurses with enough personal protective equipment and psychological support can lessen their feelings of fear. More research is needed to help understand what measures can be implemented to improve the mental health of nurses during the COVID-19 pandemic.
Galehdar N, Toulabi T, Kamran A, Heydari H. (2020) [29], Iran	Public hospital.	Intentional sample. Thirteen nurses (of whom 2 were men) who work in Intensive Care Units, Emergencies, or the Infectious Unit. They did not specify age or years of experience.	Content analysis. In-depth telephone interviews conducted be-tween March and May 2020.	The nurses who cared for people with COVID-19 in Intensive Care Units, Emergencies, or the Infectious Unit expressed fear of infection, fear of the death of patients, stress, anxiety about family separation, and fear of infecting their families.	The three main impacts of caring for people with COVID-19 manifested by nurses in Intensive Care Units, Emergencies, or in the Infectious Unit are fear, stress, and anxiety. It is necessary to counsel psychologically the nurses who care for people with COVID-19. In addition, authorities must ensure adequate precautions to prevent nurses from becoming contaminated and able to transmit the virus in their homes, improve the work environment and increase infrastructure so that nurses can focus on caring for patients. Future research should investigate proposals to improve the physical and mental performance of nurses in these circumstances.
Muz G, Erdoğan Yüce G. (2020) [30], Turkey.	Public hospital.	Intentional sample. Nineteen nurses (of whom 2 were men), between 23 and 40 years old, who work in an Intensive Care Unit and with an average work experience of 2.75 years.	Phenomenological hermeneutical design. Semi-structured interviews conducted between June and August 2020.	Caring for people with COVID-19 caused the nurses of the Intensive Care Unit anxiety and fear of being contaminated or infecting their relatives.	The main psychological impacts of caring for people with COVID-19 experienced by nurses working in Intensive Care Units are anxiety and fear of being infected or of infecting their family members. It is necessary to offer training programs periodically for professionals who work in Intensive Care Units. In addition, institutions must periodically review emergency plans. Supervising nurses must reorganize the work of nurses, considering their roles and responsibilities in their daily life. It is necessary to establish psychological support programs aimed at professionals who work with people with COVID-19. Finally, future studies should analyze this phenomenon by making worldwide comparisons.
Kackin O, Ciydem E, Aci OS, Kutlu FY. (2020) [31], Turkey.	Hospital (without specification).	Intentional sample in a snowball. Ten nurses (eight women and two men) who work in Hemodialysis, Intensive Care, Infectious Diseases, or Pneumology units caring for people with COVID-19. Average age: 29.7 years. They do not specify the time of work experience.	Descriptive Phenomenological. Semi-structured online interviews conducted between April and May 2020.	According to the nurses who worked in Hemodialysis, Intensive Care, Infectious Diseases, or Pulmonology units, caring for people with COVID-19 caused them stress, increased obsessions, increased anxiety, depressive symptoms, introversion, fear, and social isolation.	Nurses who care for people with COVID-19 in Hemodialysis, Intensive Care, Infectious Diseases, or Pulmonology units consider that they are psychologically affected, highlighting among the main impacts stress, anxiety, depression, introversion, fear, obsessions, and social isolation. It is necessary to monitor the psychological impacts experienced by nurses, implement early intervention strategies, and provide professional psychological counseling to nurses. Future research should investigate the secondary trauma that nurses may experience after caring for people with COVID-19.
Lapum J, Nguyen M, Fredericks S, Lai S, McShane J. (2020) [32], Canada	Hospital (without specification).	Intentional sample. Twenty nurses who work in acute units of hospitals in the Greater Toronto area.	Narrative analysis. Semi-structured online interviews. It is not specified when data were collected.	Caring for people with COVID-19 in acute care hospital units caused fear, apprehension, uncertainty, frustration, anger, helplessness, isolation, and sadness. Resilience was considered by the nurses to be of great help in counteracting the losses and trauma they experienced.	Nurses caring for people with COVID-19 experience psychological reactions of fear, apprehension, uncertainty, anger, helplessness, isolation, and sadness. Resilience is the main coping strategy. It is necessary to provide support to these professionals, guaranteeing the flow of information and the transparency of information, access to personal protective equipment, provide them with a guide for the prevention and control of infections, guarantee an adequate number of nurses who can cover work breaks, and provide therapeutic support in work shifts.
Arcadi P, Simonetti V, Ambrosca R, Cicolini G, Simeone S, Pucciarelli G, et al. (2021) [33], Italia	Hospitals (without specification).	Intentional sample. Twenty nurses (13 women and seven men) from different Italian hospitals. Eighteen nurses work in an Emergency Unit, one in a Home Care Unit, and another in a Medical Unit. Mean age: 27.3 years.	Hermeneutical phenomenological approach. Semi-structured online interviews (video call) conducted between March and April 2020.	Caring for hospitalized people with COVID-19 caused fear, uncertainty, and altered perception of time and space for the nurses.	The three main psychological impacts manifested by nurses who care for hospitalized people with COVID-19 are fear, uncertainty, and alterations in the perception of time and space. Psychological support and training in emergencies needs to be provided to prevent the psychological impacts of nurses caring for people with COVID-19. In addition, it is necessary to develop policies aimed at improving nursing care to ensure a better quality of care and greater patient safety in future health emergencies similar to the COVID-19 pandemic.
Tan R, Yu T, Luo K, Teng F, Liu Y, Luo J, et al. (2020) [34], China	Public hospital.	Intentional sample. Thirty nurses (does not specify the characteristics of the sample).	Content analysis. Semi-structured interview conducted between January and February 2020.	Taking care of hospitalized people with COVID-19 on the front line caused pressure, fear, anxiety, helplessness, and frustration for the nurses.	The main psychological manifestations perceived by nurses who care for hospitalized people with COVID-19 on the front line are pressure, fear, anxiety, helplessness, and frustration. Hospital managers need to strengthen psychological interventions for front line nurses as well as train them to respond effectively to emergencies. A systematic, evidence-based curriculum should be established for nurses caring for people with COVID-19. Future studies should include nurses from different units and diverse geographic locations in their sample for a better understanding of this phenomenon.
Iheduru-Anderson K. (2020) [35], United States of America	Hospitals (without specification).	Intentional sample. Twenty-eight nurses (of whom 7 were men) who work in Intensive Care, Hospitalization, or Emergency Units caring for people with COVID-19. Work experience: 3–42 years. Age: 28–65 years.	Descriptive phenomenological. Unstructured interviews via telephone or online conducted between May and June 2020.	Caring for people with COVID-19 in units that had limited access to protective equipment caused the nurses fear, a feeling of isolation, anger, a feeling of betrayal, mental overwhelm and exhaustion, and psychological pain (considering that they did not have done enough to help the patient).	Nurses caring for people with COVID-19 have psychological impacts that range from fear to anger. Nurses require ongoing mental care during the COVID-19 epidemic. Leaders and administrations must provide the physical and psychological resources to support nurses. In addition, nurse managers should monitor their nursing staff for signs of complicated grief such as anxiety, depressive symptoms, and post-traumatic stress syndrome. Future studies should examine the experiences of nurses several months and years after the pandemic is under control to see the long-term impact that nurses will have.
Cui S, Zhang L, Yan H, Shi Q, Jiang Y, Wang Q, et al. (2020) [36], China.	Hospitals (without specification).	Intentional. Twelve nurses, all women, with an average age of 34.6 years and 13.58 years of average experience, voluntarily traveled to Hubei province in China to provide support during the COVID-19 pandemic.	Content analysis. Semi-structured face-to-face interviews conducted between April and May 2020.	Nurses caring for people with COVID-19 during the pandemic experienced feelings of uncertainty, fear of infection, loneliness, stress, and sleep disturbances.	Negative experiences such as uncertainty, fear, loneliness, and sleep disorders can affect the mental health of nurses caring for people with COVID-19 during the pandemic. Front line nurses need access to supportive professional psychologists. Nurses should be encouraged to adopt self-regulatory measures, such as exercising or listening to music, as helpful tools to reduce stress.
Liu YE, Zhai ZC, Han YH, Liu YL, Liu FP, Hu DY. (2020) [37], China.	Public hospital.	Intentional sample. Fifteen nurses (5 men and 10 women) with an average age of 27.3 years and an average working life of 7.3 years who work on the front line caring for hospitalized people with COVID-19.	Content analysis. Semi-structured individual interviews conducted between January and February 2020.	Nurses caring for hospitalized people with COVID-19 on the front line experienced the following psychological impacts: fear of being infected or infecting family members, exhaustion, and stress.	Caring for people with COVID-19 on the front line causes fear, exhaustion, and stress for nurses. It is necessary to implement strategies aimed at improving the training of nurses in emergencies such as the COVID-19 pandemic. Mental health services are essential to alleviate the psychological pressure and trauma for nurses caring for people with COVID-19.
Zhang Y, Wei L, Li H, Pan Y, Wang J, Li Q, et al. (2020) [38], China.	University hospital.	Intentional sample. Twenty-three nurses, of whom 18 were women and 5 were men, with an average age of 31.5 years and 7.58 years of average work experience.	Content analysis. Semi-structured interviews conducted between February and March 2020.	The fear of being infected, loneliness derived from isolation, anxiety, depression, somatization, compulsivity, irritation, and stress were the main psychological symptoms manifested by the nurses who cared for people with COVID-19 hospitalized in the epicenter outbreak.	Nurses experience notable psychological changes during the process of caring for people hospitalized with COVID-19: fear, loneliness, depression, somatization, compulsiveness, irritation, and stress. Supervisors must pay attention to the psychological responses of their nursing staff and identify their negative emotions early. They must also provide work-related support, promoting action plans to train the nurses working in these units. Finally, they must build supportive interpersonal relationships, a strategy that would help improve the psychological health of nurses.
Fernández-Castillo RJ, González-Caro MD, Fernández-García E, Porcel-Gálvez AM, Garnacho-Montero J. (2021) [39], España.	Public hospital.	Intentional sample. Seventeen nurses (11 women and 6 men), with an average age of 40.7 and 10.2 years of average work experience who work in Intensive Care Units.	Inductive content analysis. Semi-structured online interviews (video calls) conducted in April 2020.	The main psychological impacts experienced by nurses working in Intensive Care Units caring for people with COVID-19 were fear, anxiety, frustration, and isolation.	Nurses caring for people with COVID-19 in Intensive Care Units experience fear, anxiety, frustration, and isolation, which affects nursing care. Healthcare leaders must implement a model of care centered on the patient and nurse, where improvement benefits both parties. They must also provide an optimal work environment, especially in anticipation of the mental health and safety issues that nurses may experience.

**Table 3:** Evaluation of the quality of the studies with the CASP tool for qualitative studies.

Authors	1. Was There a Clear Statement of the Aims of the Research?	Is a Qualitative Methodology Appropriate?	Was the Research Design Appropriate to Address the Aims of the Research?	Was the Recruitment Strategy Appropriate to the Aims of the Research?	Was the Data Collected in a Way that Addressed the Research Issue?	Has the Relationship between Researcher and Participants Been Adequately Considered?	Have Ethical Issues Been Taken into Consideration?	Was the Data Analysis Sufficiently Rigorous?	Is there a Clear Statement of Findings?	How Valuable Is the Research?
Karimi Z, Fereidouni Z, Behnammoghadam M, Alimohammadi N, Mousavizadeh A, Salehi T, et al. (2020) [27]	YES	YES	YES	YES	YES	YES	YES	YES	YES	YES
Galehdar N, Kamran A, Toulabi T, Heydari H. (2020) [28]	YES	YES	YES	YES	YES	YES	YES	CANNOT TELL	YES	YES
Galehdar N, Toulabi T, Kamran A, Heydari H. (2020) [29]	YES	YES	YES	YES	YES	YES	YES	YES	YES	YES
Muz G, Erdoğan Yüce G. (2020) [30]	YES	YES	YES	YES	YES	YES	YES	YES	YES	YES
Kackin O, Ciydem E, Aci OS, Kutlu FY. (2020) [31]	YES	YES	YES	YES	YES	YES	YES	YES	YES	YES
Lapum J, Nguyen M, Fredericks S, Lai S, McShane J. (2020) [32]	YES	YES	YES	YES	YES	NO	YES	YES	YES	YES
Arcadi P, Simonetti V, Ambrosca R, Cicolini G, Simeone S, Pucciarelli G, et al. (2021) [33]	YES	YES	YES	YES	YES	CANNOT TELL	YES	YES	YES	YES
Tan R, Yu T, Luo K, Teng F, Liu Y, Luo J, et al. (2020) [34]	YES	YES	YES	YES	YES	CANNOT TELL	YES	YES	YES	YES
Iheduru-Anderson K. (2020) [35]	YES	YES	YES	YES	YES	YES	YES	YES	YES	YES
Cui S, Zhang L, Yan H, Shi Q, Jiang Y, Wang Q, et al. (2020) [36]	YES	YES	YES	YES	YES	YES	YES	YES	YES	YES
Liu YE, Zhai ZC, Han YH, Liu YL, Liu FP, Hu DY. (2020) [37]	YES	YES	YES	YES	YES	YES	YES	YES	YES	YES
Zhang Y, Wei L, Huanting L, Pan Y, Wang J, Qianquian L, et al. (2020) [38]	YES	YES	YES	YES	YES	YES	YES	YES	YES	YES
Fernández-Castillo RJ, González-Caro MD, Fernández-García E, Porcel-Gálvez AM, Garnacho-Montero J. (2021) [39]	YES	YES	YES	YES	YES	YES	YES	YES	YES	YES
